# Detection of anti‐NS1 antibodies after pandemic influenza exposure: Evaluation of a serological method for distinguishing H1N1pdm09 infected from vaccinated cases

**DOI:** 10.1111/irv.12712

**Published:** 2020-01-19

**Authors:** Anna Hayman Robertson, Milada Mahic, Miloje Savic, Gro Tunheim, Olav Hungnes, Lill Trogstad, Walter Ian Lipkin, Siri Mjaaland

**Affiliations:** ^1^ Division of Infection Control and Environmental Health Norwegian Institute of Public Health Oslo Norway; ^2^ Center for Infection and Immunity Mailman School of Public Health Columbia University New York NY USA; ^3^ KG Jebsen Center for Influenza Vaccine Research Oslo Norway; ^4^ WHO National Influenza Centre Oslo Norway; ^5^ Present address: GSK Biologicals S.A. Wavre Belgium

**Keywords:** H1N1pdm09, influenza, NS1, pandemic, serology, vaccination

## Abstract

**Background:**

Reliable exposure information is crucial for assessing health outcomes of influenza infection and vaccination. Current serological methods are unable to distinguish between anti‐hemagglutinin (HA) antibodies induced by infection or vaccination.

**Objectives:**

We aimed to explore an alternative method for differentiating influenza infection and vaccination.

**Methods:**

Sera from animals inoculated with influenza viruses or purified H1N1pdm09 HA were obtained. Human samples were selected from a pregnancy cohort established during the 2009 H1N1 pandemic. Unvaccinated, laboratory‐confirmed cases (N = 18), vaccinated cases without influenza‐like‐illness (N = 18) and uninfected, unvaccinated controls (N = 18) were identified based on exposure data from questionnaires, national registries and maternal hemagglutination inhibition (HI) titres at delivery. Animal and human samples were tested for antibodies against the non‐structural protein 1 (NS1) and HA from H1N1pdm09, using a Luciferase Immunoprecipitation System (LIPS).

**Results:**

Anti‐NS1 H1N1pdm09 antibodies were detected in sera from experimentally infected, but not from vaccinated, animals. Anti‐HA H1N1pdm09 antibodies were detectable after either of these exposures. In human samples, 28% of individuals with laboratory‐confirmed influenza were seropositive for H1N1pdm09 NS1, whereas vaccinated cases and controls were seronegative. There was a trend for H1N1pdm09 NS1 seropositive cases reporting more severe and longer duration of symptomatic illness than seronegative cases. Anti‐HA H1N1pdm09 antibodies were detected in all cases and in 61% of controls.

**Conclusions:**

The LIPS method could differentiate between sera from experimentally infected and vaccinated animals. However, in human samples obtained more than 6 months after the pandemic, LIPS was specific, but not sufficiently sensitive for ascertaining cases by exposure.

## 1 INTRODUCTION

Influenza is an acute respiratory disease caused by influenza type A or B viruses, associated with increased rates of hospitalization and death in susceptible individuals.^1^, ^2^ Annual vaccination against seasonal influenza is recommended to high‐risk groups by the World Health Organization (WHO).^3^ In 2009, a novel H1N1 influenza A virus emerged causing a global pandemic with significant morbidity and mortality.^1^, ^4^, ^5^ In Norway, a national campaign with an adjuvanted, monovalent vaccine (Pandemrix, GSK Biologicals) coincided with the pandemic peak.^6^, ^7^ The pre‐pandemic prevalence of antibodies at protective titres was low in the Norwegian population.^8^

To disentangle the role of pandemic influenza infection and vaccination on different health outcomes, reliable exposure information is essential. The gold standard for the direct detection of influenza infection is laboratory identification of influenza viral RNA by reverse transcription‐polymerase chain reaction (RT‐PCR) or virus culture from respiratory samples.^9^ Alternative methods include other nucleic acid based technologies or the detection of viral antigen by, for example immunofluorescence assays. Direct detection requires sampling during the acute phase of infection, preferably 3‐4 days within symptom onset,^9^ and is typically applied to support clinical management of severe cases. For mild cases, a clinical diagnosis based on symptoms may suffice,^9^ or medical care may not be sought; moreover, a large proportion of infected individuals remain asymptomatic.^10^, ^11^

Consequently, most cases of influenza infection remain undetected,^6^, ^7^ underscoring the need for other sources of information, such as questionnaires, health registries and biological specimens, for ascertaining or estimating the infection status of an individual. In prospective studies, direct laboratory testing requires active screening of participants, which is labour intensive and expensive, and typically limited to clinical vaccine trials. Indirect detection by the hemagglutination inhibition (HI) assay has traditionally been the main tool for identification of influenza infections, whereby influenza anti‐hemagglutinin (HA) specific antibodies are measured.^12^ An increase in HI titre (typically fourfold over a base level) indicates an infection. However, the assay cannot distinguish between antibodies induced from infection and vaccination, as inactivated vaccines, like infection, also induce antibody responses against the viral HA surface antigen. In contrast, antibodies against the non‐structural protein‐1 (NS1) are only induced upon active viral replication during infection.^13^

The Luciferase Immunoprecipitation System (LIPS) has been used as a rapid serological assay to detect viral agents^14^ and vaccine responses.^15^ We sought to evaluate whether the LIPS method, by measuring anti‐NS1 antibody levels, could discriminate between influenza H1N1pdm09 infection and vaccination, firstly in control sera from experimentally immunized animals, and secondly in samples from pregnant women in a cohort established during the 2009 pandemic in Norway.

## 2 MATERIAL AND METHODS

### 2.1 Animal antisera

Ferret antisera to Influenza Viruses A/Brisbane/59/2007 (H1N1), #FR‐388, A/Brisbane/10/2007 (H3N2), #FR‐389, A/Wisconsin/15/2009 (H3N2), #FR‐445, A/New Caledonia/20/1999 (H1N1), #FR‐955, A/Fujian/411/2002 (H3N2), #FR‐1264, B/Florida/4/2006, #FR‐391, were obtained through the Influenza Reagent Resource (IRR), Influenza Division, WHO Collaborating Center for Surveillance, Epidemiology and Control of Influenza, Centers for Disease Control and Prevention, Atlanta, GA, USA. They were derived from ferrets intranasally infected with the respective specified viruses (typically 10^6^ CEID_50_), and sera collected 15‐33 days post‐infection. 2014‐2015 WHO Antiserum, Influenza A(H1N1)pdm09 Reference Sheep Antiserum, #FR‐1213, and the Influenza Normal Control Sheep Serum, #FR‐49, were also obtained from the IRR. #FR‐1213 was prepared in sheep by multiple intramuscular injections with purified HA,^16^ equivalent to vaccination. The #FR‐49 sera served as a negative control. A positive control antisera was obtained from ferrets experimentally infected with 10^4^ plaque forming units of A/California/04/2009 (H1N1)pdm09 and antisera after 21 days, kindly provided by Dr RA Albrecht, the Icahn School of Medicine, Mount Sinai.

### 2.2 Study population

Human plasma samples were obtained from the Norwegian Influenza Pregnancy Cohort Study (NorFlu) established during the A/H1N1 pandemic in 2009, as described previously.^17^ The study was approved by the Regional Committees for Medical and Health Research Ethics South East (2009/2165). Participants completed a questionnaire during pregnancy indicating by check box if, and if relevant, when they received the pandemic vaccine, if they had had influenza or experienced any of several symptoms of influenza during the current pregnancy and pandemic period or had taken antivirals against influenza.

Blood samples were collected from participants at delivery. Neutralizing anti‐HA antibody levels were measured using the HI assay with the pandemic vaccine virus NYMC X‐179A as described previously.^17^ If there was no HI at the highest serum concentration (1:10 dilution), the titre was assigned a value of 5 for calculation purposes.

#### 2.2.1 Linkage to National Health Registries

Additional data were obtained from national registries through linkage with the unique personal identification number assigned to all residents of Norway. Information about the pregnancy was obtained from the Medical Birth Registry of Norway (MBRN). Records of pandemic vaccination against H1N1pdm09 influenza and cases of laboratory RT‐PCR‐confirmed H1N1pdm09 influenza were retrieved from the Norwegian Immunization Registry (SYSVAK) and the Norwegian Surveillance system for Communicable Diseases (MSIS), respectively. Records on clinical influenza diagnosis by primary care physicians (R‐80, International Classification of Primary Care, Second edition, ICPC‐2) were obtained from the Directorate of Health's reimbursement database (KUHR). In Norway, the main pandemic period occurred between 1 October 2009 and 31 December 2009 peaking in early November.^6^, ^7^ Due to limited laboratory capacity and restrained testing during the pandemic, MSIS‐reported cases only represent a fraction of the real number of infected cases and are biased towards severe cases. Registration of pandemic vaccination was mandatory and less prone to detection bias.^18^

#### 2.2.2 Selection criteria

Three groups were selected among NorFlu participants, who were pregnant during the pandemic peak:

*“LCI cases,”* that is unvaccinated, laboratory‐confirmed influenza (LCI) cases (N = 18) included women who were registered in MSIS with a laboratory RT‐PCR‐confirmed H1N1pdm09 infection during the pandemic peak, had an H1N1pdm09 HI titre ≥20 at delivery, with no record of pandemic vaccination in SYSVAK, nor self‐reported pandemic vaccination.

*“Vaccinated cases”* without ILI (influenza‐like‐illness) (N = 18) included women who had a record of one dose of pandemic vaccine in SYSVAK, no record in MSIS of a H1N1pdm09 LCI, no record of a clinical influenza diagnosis (R80), no self‐reported ILI or ILI symptoms and no use of antiviral medication.

“Controls” (N = 18) included women with no record in MSIS of a H1N1pdm09 LCI, no record of a clinical diagnosis (R‐80), nor pandemic vaccination in SYSVAK, no self‐reported pandemic vaccination, ILI or ILI symptoms, and no use of antiviral medication, and had a H1N1pdm09 HI titre < 10 at delivery.

### 2.3 Luciferase Immunoprecipitation System (LIPS)

The LIPS technology, based on luciferase‐tagged antigens produced in mammalian Cos1 cells, was used to screen the samples for antibodies against H1N1pdm09 NS1 and HA. HA and NS1 of A/California/07/09 (H1N1)pdm09 were amplified by RT‐PCR and subcloned into a mammalian Renilla luciferase (Ruc) expression vector (pREN2) to generate Ruc‐HA and Ruc‐NS1 C‐terminal fusion protein constructs respectively. Construct identity and integrity was confirmed by DNA sequencing. Cos1 cells (10^6^/10 cm round plate) were transiently transfected with 2 µg of the Ruc‐NS1 or Ruc‐HA construct. Cell extracts containing the recombinant proteins (CERP) were prepared as described previously.^14^, ^19^

### 2.4 Measurement of antibody levels using the LIPS system

All sera or plasma samples were diluted 1:10 in buffer A (50 mM Tris, pH 7.5, 100 mM NaCl, 5 mM MgCl_2_, 1% Triton X‐100) in a 96‐deep‐well polypropylene microtiter plate and shaken extensively (1‐2 hours) on a rotator platform. About 10 µL of diluted sample was further mixed with 40 µL of buffer A and 50 µL of CERP diluted in buffer A to the equivalent of 2 × 10^6^ light units (LU) (to ensure standardized quantities of NS1/HA antigen across runs) and incubated in a 96‐well polypropylene plate on a rotary shaker for 1 hour at room temperature. Five µL of 30% suspension of Ultralink protein A/G beads (Thermo Scientific Pierce), resuspended in buffer A, were added to a 96‐well filter plate. Sample mixes were transferred to the filter plate containing Ultralink protein A/G beads and incubated on a rotary shaker for one hour at room temperature to pull down IgG antibody‐Ruc‐HA or antibody‐Ruc‐NS1 complexes. Beads with bound protein complexes were washed 3 times in Buffer A, and the plate was blotted dry.^19^

Coelenterazine luciferase substrate was prepared using the Promega Renilla substrate kit as described by the manufacturer. Fluorescence (LU) was measured on a Berthold Centro LB 960 plate reader. Estimates represent the average of at least two independent measurements. The mean fold increase for each sample was estimated relative to the negative control serum for each run. Experiments with animal sera were corrected for background fluorescence.

To define H1N1pdm09 seropositivity according to LIPS, a simple statistically based cut‐off was derived for the NS1 and HA antigens, respectively, from the mean value of the signal from the negative control plus five standard deviations (SDs). A cut‐off using the mean plus three SDs was also tested, but resulted in reduced specificity and no increase in sensitivity. NS1 or HA serostatus was used to determine the specificity, sensitivity, positive predictive value (PPV) and negative predictive value (NPV) for the detection of infection (ie LCI cases vs controls) and for distinguishing infected from vaccinated cases (ie LCI cases vs vaccinated cases).

### 2.5 Time since exposure

Time between exposure and blood sampling was the interval in days from exposure (date of LCI or vaccination) and date of birth (sampling). For the controls, exposure was defined as the date of the pandemic peak (2 November 2009).^6^

### 2.6 Statistical analysis

Comparisons of continuous outcomes were performed using a *t* test. (comparisons of categorical outcomes were not performed due to small sample size). Analyses were carried out using Stata/S 14.0 (StataCorp LCC), Microsoft Excel (Microsoft Corporation) and GraphPad Prism 8 (GraphPad Software, Inc).

## 3 RESULTS

### 3.1 LIPS detection of anti‐H1N1pdm09 antibodies against NS1 and HA in sera from experimentally inoculated animals

**Figure 1:**
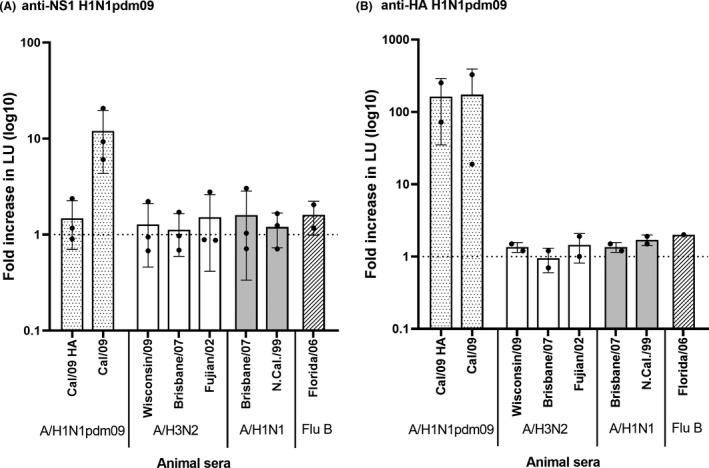
Antibody levels against H1N1pdm09 NS1 (A) and HA (B) in animal sera with known exposure status, measured by LIPS. The panel included a positive control from ferrets inoculated with H1N1pdm09 virus (“Cal 09”), sera from sheep immunized with purified H1N1pdm09 HA (“Cal/09 HA”), and from ferrets intranasally infected with pre‐pandemic strains. Antibody levels are shown as the mean fold increase in luminescence (light units, LU) relative to the negative control. Error bars indicate the standard deviation. Data points are shown for each run

A panel of antisera from ferrets inoculated with different strains of influenza virus was tested for antibodies against the H1N1pdm09 viral NS1 protein by LIPS (Figure 1A). Sera from ferrets inoculated with H1N1pdm09 virus were included as a positive control for pandemic infection (“Cal/09”). A higher level of anti‐NS1 antibodies was detected in the positive control compared to the negative control, representing a 12.0 (SD ± 7.7) fold increase in LU (*P*‐value of .077). The increase varied considerably across measurements for the same sample (eg ranging from 6.1 to 20.6 fold). In contrast, a minimal increase in LU (1.5 ± SD 0.8) was measured in the antisera against purified H1N1pdm09 HA mimicking vaccination (“Cal/09 HA”, control sera without anti‐NS1 antibodies), and other non‐pandemic viruses circulating prior to the pandemic (ranging from 1.1 to 1.6).

The animal antisera were subsequently tested for the presence of antibodies against the H1N1pdm09 HA protein (Figure 1B). As expected, anti‐HA antibodies were detected in sera raised against the pandemic virus (“Cal/09”) or against the H1N1pdm09 HA antigen (“Cal/09 HA”), but not in the antisera against other, non‐H1N1pdm09, virus strains. The relative level of H1N1pdm09 specific antibodies in the positive control was much higher for the anti‐HA than for anti‐NS1 response (approximately 170‐ and 12‐fold increase, respectively).

### 3.2 LIPS detection of anti‐H1N1pdm09 NS1 and HA antibodies in human laboratory‐confirmed infection and in vaccination

**Figure 2:**
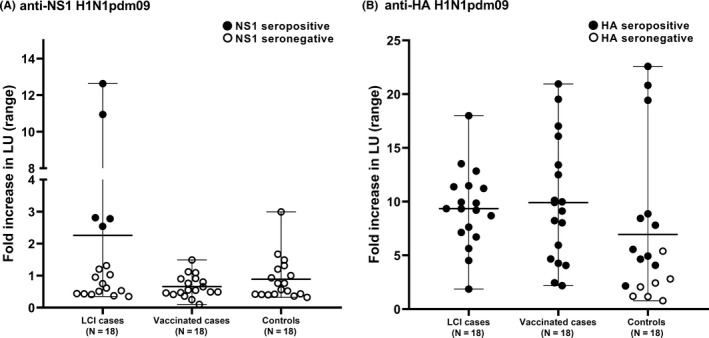
Antibody levels against H1N1pdm09 NS1 (A) and HA (B) measured by LIPS in human plasma from laboratory‐confirmed influenza (LCI) cases, vaccinated cases and controls. Antibody levels are shown as the mean fold increase in luminescence (light units, LU) relative to the negative control. Error bars indicate the range for each group. H1N1pdm09 seropositive and negative samples are shown in closed and open circles, respectively. (Note seropositivity is defined according to a statistically based cut‐off value for each individual run, and not by fold increase.)

Next, we tested the LIPS platform on plasma from 18 pregnant, unvaccinated women who had laboratory RT‐PCR‐confirmed H1N1pdm09 infection (“LCI cases”), compared to 18 pregnant vaccinated women without ILI (“vaccinated cases”), and 18 pregnant, uninfected, unvaccinated women (“controls”) (Figure 2). The mean fold increase of the anti‐NS1 antibody response was higher in the LCI cases compared to the controls (2.3 SD ± 3.6 vs 0.9 ± SD 0.7, respectively, *P*‐value of .12) (Figure 2A). Similarly, the anti‐NS1 antibody levels were higher in the LCI cases compared to the vaccinated cases (2.3 ± SD 3.6 vs 0.7 ± SD 0.4 fold increase, respectively, *P*‐value of .07) (Figure 2A). The range in antibody levels within each group was large.

Using a statistically defined cut‐off, only 5/18 (28%) LCI cases were NS1 seropositive; the remaining cases and controls were NS1 seronegative. In comparison with the vaccinated and control cases, the fold increase for the NS1 seropositive LCI cases was significant (*P*‐values of <.001 and .001, respectively). The specificity of the NS1 LIPS method for distinguishing H1N1pdm09 LCI cases from controls was high (100%), but the sensitivity was only 28% (Table 1). The positive predictive value (PPV) was high (100%), and the negative predictive value (NPV) was moderate (58%). Similarly, the method could distinguish LCI cases from vaccinated cases with high specificity (100%), but low sensitivity (28%).

**Table 1:** Specificity, sensitivity, positive predictive values (PPV) and negative predictive values (NPV) for the detection of infection and for distinguishing infected from vaccinated cases

Comparison	Infected/vaccinated^a^	N	Positive	Negative	Sensitivity	Specificity	PPV	NPV
NS1 seropositive								
Infection	−/−	18	0	18				
	+/−	18	5	13	28%	100%	100%	58%
Infection vs Vaccination	−/+	18	0	18				
	+/−	18	5	13	28%	100%	100%	58%
HA seropositive								
Infection	−/−	18	11	7				
	+/−	18	18	0	100%	39%	62%	100%
Infection vs Vaccination	−/+	18	18	0				
	+/−	18	18	0	100%	0%	50%	0%

Note

Estimates are based on anti‐H1N1pdm09 NS1 and HA serostatus according to LIPS.

a The “+” and “−” signs reflect the case definitions as follows: Uninfected, unvaccinated controls: “−/−,” LCI, unvaccinated cases: “+/−,” and Vaccinated cases without ILI: “−/+”.

In contrast, the mean anti‐HA antibody level was similar for the LCI (9.4 ± 3.6) and vaccinated cases (9.9 ± SD 5.7), and only marginally higher than for the controls (7.0 ± SD 7.0) (Figure 2B). All the LCI and vaccinated cases were HA seropositive (Table 1). Among the controls, 11/18 (61%) were HA seropositive.

### 3.3 Comparison between H1N1pdm09 specific anti‐NS1, anti‐HA and HI antibody titres

In the LCI samples, the mean antibody level was higher for the anti‐HA response compared to the anti‐NS1 response (9.4 ± SD 3.6 vs 2.3 ± SD 3.6, *P* < .001), and the level of anti‐NS1 antibody was higher in samples with an HI > 40 (12.7 ± SD 3.3, N = 5) compared to those with an HI ≤ 40 (8.1 ± SD 2.9, N = 13). The vaccinated cases were all HA seropositive according to LIPS, but 4/18 (22%) cases had undetectable HI titres (HI < 10). The correlation between the HI titres and anti‐HA levels for the LCI cases (r = 0.447, *P*‐value of .063) and vaccinated cases (*r* = .484, *P*‐value of .042) was moderate, but less apparent for the anti‐NS1 antibody levels (LCI cases) (Figure S1).

### 3.4 Sensitivity of anti‐H1N1pdm09 NS1 antibody detection and association with severity of pandemic illness and time since exposure

To explore possible explanations for the poor sensitivity of anti‐H1N1pdm09 specific NS1 antibody detection in human LCI samples, data on HI titre, time since exposure, medical attention and self‐reported severity of illness were compared for the NS1 seropositive (N = 5) and seronegative (N = 13) LCI cases (Table S1). The sample size was too small for statistical analysis, but several trends were observed. The geometric mean HI titre (GMT) in the NS1 seropositive samples was higher than in the seronegative samples (52.8 (95%CI 24.4‐114.0) vs 30.6 (95%CI 22.2‐42.3)), and a higher proportion of NS1 seropositive cases sought medical attention multiple times during the course of their ILI episode (60% vs 38%). NS1 seropositive women appeared to feel more ill, be ill for a longer period of time, and more frequently report ILI specific symptoms.

The mean time between exposure and sampling was similar between the NS1 seropositive and negative cases (Table S1), and between the LCI cases −215 days (95%CI 199‐232) and the vaccinated cases −214 days (95%CI 187‐240). The interval was slightly longer for the controls −254 days (95%CI 236‐272).

### 3.5 Screening of H1N1pdm09 HI seropositive, asymptomatic, unvaccinated cases

Based on our findings that severity of illness may affect NS1 antibodies, we wished to explore if anti‐H1N1pdm09 NS1 specific antibodies were more difficult to detect after asymptomatic infection compared to symptomatic infection. A high HI titre (>40) against H1N1pdm09 was used as an indication of infection, based on the low pre‐pandemic titres in the population.^8^ Ten unvaccinated cases with high HI titres (HI GMT 98.5, 95%CI 70.5‐137.6), who did not seek medical attention (ie lacking a LCI or clinical diagnosis), nor self‐reported ILI, were tested. Only 1/10 cases were NS1 seropositive. The mean time since exposure was 246 days (95%CI 182‐296).

## 4 DISCUSSION

In this study, we have evaluated LIPS as a method to distinguish between influenza pandemic H1N1/2009 infection and vaccination in human and animal control samples. In sera from laboratory animals with no prior exposure history to influenza, we demonstrated that the method could detect anti‐NS1 H1N1pdm09 specific antibodies in experimentally infected animals, but not after inoculation with purified H1N1pdm09 HA.

No anti‐NS1 H1N1pdm09 response was detected in human controls or vaccinated cases, implying high specificity (100%) of the method. However, only 28% of the laboratory RT‐PCR‐confirmed H1N1pdm09 influenza cases had detectable anti‐NS1 H1N1pdm09 specific antibodies. In contrast, all vaccinated and infected individuals were LIPS H1N1pdm09 HA seropositive. A large proportion (61%) of the controls (HI‐negative) were also LIPS HA seropositive, likely due to the LIPS method being more sensitive than the HI assay. Both LIPS and ELISA^20^ based assays measure all binding antibodies, in contrast to the HI assay that only measures antibodies that prevent virus attachment to red blood cells. While the LIPS HA seropositive controls may represent true subclinical infections,^10^, ^11^ cross‐reactivity of anti‐H1 antibody to previous non‐H1N1pdm09 infections is possible.^8^

The main strength of this study was access to human samples from a well‐characterized cohort, combining national registry data, self‐reported questionnaire data and HI titre data.^17^, ^21^, ^22^ During the pandemic, it was mandatory to report LCI and vaccination to the national registries in Norway. The registry data validated the participant‐completed questionnaires; all of the MSIS‐registered LCI cases self‐reported seeking medical attention and taking a nasal swab. Likewise, all vaccinated cases were registered in the national immunization registry and self‐reported vaccination. Combining data sources enabled the selection of uninfected (HI < 10), unvaccinated controls, though we cannot exclude the possibility of subclinical infection, since the MSIS database was subject to underreporting, and low HI titres could have waned to undetectable levels. The misclassification of vaccination status is unlikely,^18^ as supported by the corresponding HI titre data. Furthermore, pregnant women are generally more vigilant and likely to remember receiving a new pandemic vaccine.

The main limitation of this study was the considerable time between human pandemic infection (>6 months) and sampling, during which antibody titres against NS1 and HA are likely to have waned,^17^ and in some samples probably below the limit of detection. This is in contrast to the control animal sera, which were collected 2‐4 weeks post‐infection. Conceivably, some LCI cases could also have responded with anamnestic anti‐NS1 antibodies that react better to the NS1 of non‐H1N1pdm09 viruses. To our knowledge, there are few studies on the longevity of the anti‐influenza NS1 antibody response. In chickens, the response peaked 3 weeks post‐infection and rapidly decreased by week 5,^23^ implying a short‐lived response. Kinetics could be dependent on species^24^ and influenza strain. Further, we measured a higher fold increase in antibody levels against HA relative to NS1. Structural viral (surface) antigens are probably more abundant and accessible and induce more antibodies than accessory/regulatory proteins.^14^ Pregnancy may also alter the antibody response, though studies on vaccination in pregnancy do not suggest this.^25^

Others have applied serological methods for differentiating influenza infection and vaccination through detection of anti‐NS1 antibodies, in horses,^26^ chickens^23^ and humans.^27^, ^28^ In chickens, detection was more successful after challenge with highly pathogenic avian influenza (HPAI) compared to low pathogenic avian influenza (LPAI) implying a correlation between antibody response and severity of illness. We made a similar observation, to be interpreted with caution due to the small sample size of the LCI group. However, previous analysis on a larger sample size from the same pregnancy cohort supported a correlation between HI titre and disease severity.^17^

Another limitation was the large degree of variation in LU values between measurements, suggesting the LIPS method is more appropriate for defining serostatus than antibody quantification, at least for H1N1pdm09. It was not sensitive enough for screening the remaining participants for H1N1pdm09 infection in our pregnancy cohort where LCI data are lacking. For seasonal influenza, the confirmation of infection cannot be based on the detection of the anti‐NS1 response by the LIPS method alone, due to the co‐circulation of strains and the conserved nature of NS1. Supplementary methods are necessary for strain identification, such as the HI assay, utilizing surveillance data on circulating strains.^29^

Future work is warranted to explore the sensitivity and specificity of anti‐NS1 antibody detection during the acute phase of infection and at subsequent time intervals, for different subtypes and strains, and in samples from vaccinated individuals. This is important to assess the applicability of the LIPS method for the detection of recent or previous influenza infections and for distinguishing infected and vaccinated cases. Other viral antigens not present in the vaccine may also be considered for the LIPS assay, such as the NS2 protein, in combination with NS1, to improve sensitivity.

In conclusion, we showed that the LIPS method could distinguish between pandemic H1N1pdm09 infection and vaccination in experimentally inoculated animals. However, in human cases, although the method was specific, it was not sensitive for detecting pandemic H1N1pdm09 infection, at least not in samples obtained more than 6 months after the pandemic.
