# Supplementary material for: Detection of anti‐NS1 antibodies after pandemic influenza exposure: Evaluation of a serological method for distinguishing H1N1pdm09 infected from vaccinated cases
Source: Influenza Other Respir Viruses. 2020 Jan 19;14(3):294–301. doi: 10.1111/irv.12712 (PMC7182603; doi:10.1111/irv.12712)

**Figure S1:** Comparison between H1N1pdm09 antibody titers measured according to LIPS and the HI assay in human laboratory confirmed (LCI) cases (circles) and vaccinated cases (triangles). A) Fold increase in anti-NS1 antibody levels versus HI titer. B) Fold increase in anti-HA antibody levels versus HI titer. The correlation coefficient (Spearman’s r) and corresponding p-value are shown for each comparison.

A) Fold increase in anti-NS1 levels versus HI titer B) Fold increase in anti-HA levels versus HI titer


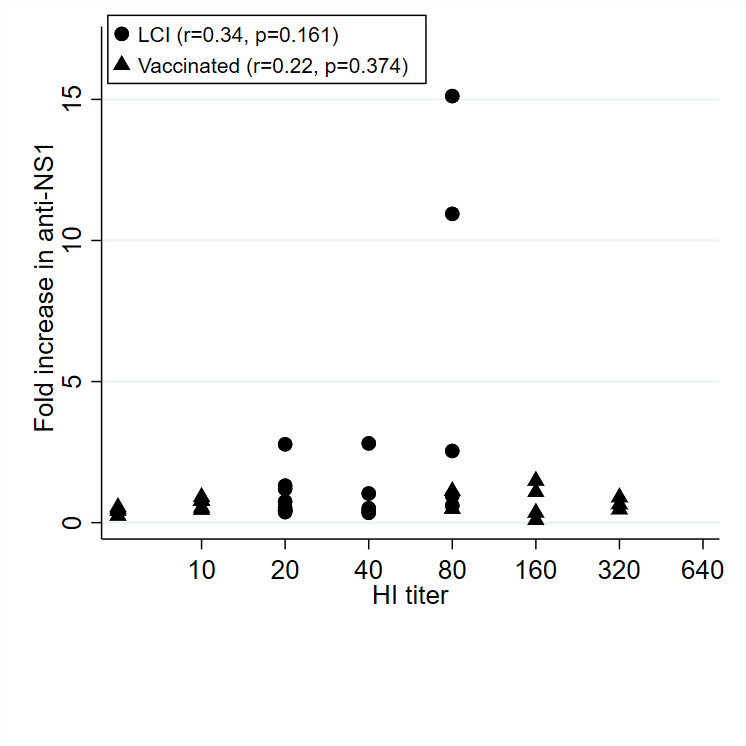

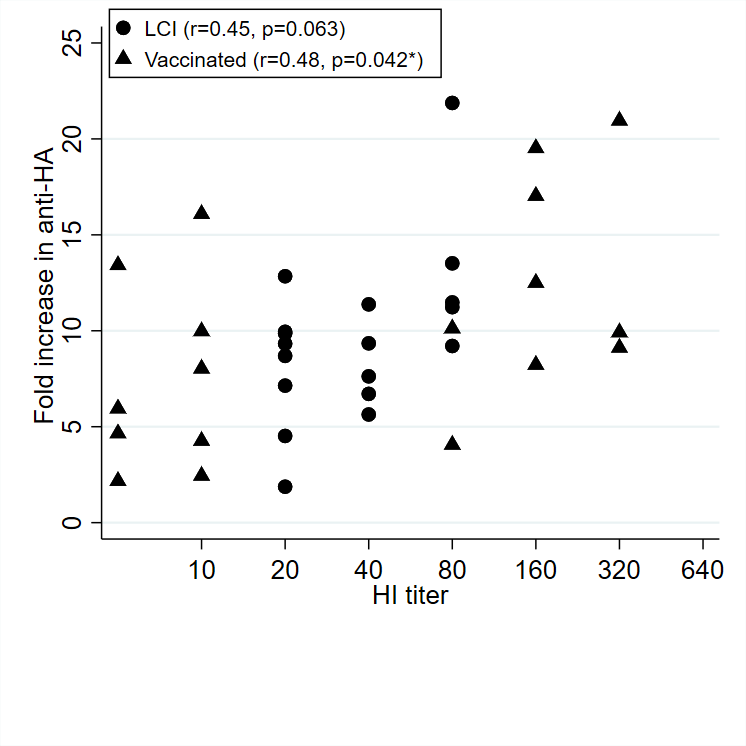

Supplement: Supplementary file 1 [file IRV-14-294-s001.docx]
