# Supplementary material for: Detection of anti‐NS1 antibodies after pandemic influenza exposure: Evaluation of a serological method for distinguishing H1N1pdm09 infected from vaccinated cases
Source: Influenza Other Respir Viruses. 2020 Jan 19;14(3):294–301. doi: 10.1111/irv.12712 (PMC7182603; doi:10.1111/irv.12712)
Supplement: Supplementary file 2 [file IRV-14-294-s002.docx]

**SUPPLEMENTARY INFORMATION**

**Table S1**. Comparison of H1N1pdm09 NS1 seropositive (N=5) and seronegative (N=13) laboratory-confirmed influenza (unvaccinated) cases with respect to HI titres, time since exposure, medical attention, and self-reported symptoms. (Note that NS1 seropositivity is defined according to the LIPS method).

| **Variable** | **NS1 seropositive**  **(N=5)** | **NS1 seronegative**  **(N=13)** |
| --- | --- | --- |
| Anti-NS1 H1N1pdm01 level, LU | 578 027 ± 98 804 | 150 296 ± 5628 |
| HI titre, GMT† (95%CI) | 52.8 (24.4- 114.0) | 30.6 (22.2- 42.3) |
| Proportion with HI titre ≥20 | 80 % | 46 % |
| Time between infection and sampling in days, mean (95% CI) | 216 (170.5-262.2) | 212 (197.7-226.3) |
| Medical attention‡ | 4/5 (80%) | 10/13 (77%) |
| Medical attention^§^ > 1 during illness | 3/5 (60%) | 5/13 (38%) |
| **Self-reported illness** | | |
| Fever¶ | 5/5 (100%) | 13/13(100%) |
| CDC-case definition of influenza like illness (ILI) ^&^ | 5/5 (100%) | 13/13(100%) |
| Length of illness > 5 days | 3/3 (100%)^#^ | 5/11 (45%)^#^ |
| Severity of illness, %: | Quite ill/very ill: 5/5 (100%) | Quite ill/very ill: 9/13 (69%) |
| > 6 symptoms | 4/5 (80%) | 8/13 (62%) |
| Headache | 4/5 (80%) | 10/13 (77%) |
| Cough | 5/5 (100%) | 10/13 (77%) |
| Stuffy nose | 5/5 (100%) | 8/13 (62%) |
| Sore throat | 5/5 (100%) | 10/13 (77%) |
| Pneumonia | 0/5 (0%) | 1/13 (8%) |
| Joint pain | 2/5 (40%) | 9/13(69%) |
| Muscle pain | 5/5 (100%) | 8/13 (62%) |
| Out of breath | 4/5 (80%) | 8/13 (62%) |
| Chest pain | 2/5 (40%) | 3/13 (23%) |
| Vomiting /diarrhea | 1/5 (20%) | 4/13(31%) |

^+^GMT, geometric mean titre

Minimum one record (‡) or more than one record (^§^) of clinical diagnosis by primary care (R-80, ICPC-2) during the pandemic peak.

^¶^Extracted from questionnaire data: “fever > 39°C,” “fever < 39°C,” or “unmeasured fever”.

^&^Fever >37.8°C, cough and/or a sore throat in the absence of another known cause (https://www.cdc.gov/flu/weekly/overview.htm#Outpatient). “Fever >37.8°C” was replaced with the combined definition of “fever”.

^#^Data missing
